# Shedding light: a phylotranscriptomic perspective illuminates the origin of photosymbiosis in marine bivalves

**DOI:** 10.1186/s12862-020-01614-7

**Published:** 2020-05-01

**Authors:** Jingchun Li, Sarah Lemer, Lisa Kirkendale, Rüdiger Bieler, Colleen Cavanaugh, Gonzalo Giribet

**Affiliations:** 1 Ecology and Evolutionary Biology, University of Colorado Boulder, Boulder, USA; 2 Museum of Natural History, University of Colorado Boulder, Boulder, USA; 3 Organismic and Evolutionary Biology, Harvard University, Cambridge, USA; 4 University of Guam Marine Laboratory, Mangilao, Guam; 5 Museum of Comparative Zoology, Harvard University, Cambridge, USA; 6 Department of Aquatic Zoology, Western Australian Museum, Welshpool, Australia; 7 Negaunee Integrative Research Center, Field Museum of Natural History, Chicago, USA

**Keywords:** Photosymbiosis, Tridacinae, Fraginae, Symbiodiniaceae, Reef habitat

## Abstract

**Background:**

Photosymbiotic associations between metazoan hosts and photosynthetic dinoflagellates are crucial to the trophic and structural integrity of many marine ecosystems, including coral reefs. Although extensive efforts have been devoted to study the short-term ecological interactions between coral hosts and their symbionts, long-term evolutionary dynamics of photosymbiosis in many marine animals are not well understood. Within Bivalvia, the second largest class of mollusks, obligate photosymbiosis is found in two marine lineages: the giant clams (subfamily Tridacninae) and the heart cockles (subfamily Fraginae), both in the family Cardiidae. Morphologically, giant clams show relatively conservative shell forms whereas photosymbiotic fragines exhibit a diverse suite of anatomical adaptations including flattened shells, leafy mantle extensions, and lens-like microstructural structures. To date, the phylogenetic relationships between these two subfamilies remain poorly resolved, and it is unclear whether photosymbiosis in cardiids originated once or twice.

**Results:**

In this study, we establish a backbone phylogeny for Cardiidae utilizing RNASeq-based transcriptomic data from Tridacninae, Fraginae and other cardiids. A variety of phylogenomic approaches were used to infer the relationship between the two groups. Our analyses found conflicting gene signals and potential rapid divergence among the lineages. Overall, results support a sister group relationship between Tridacninae and Fraginae, which diverged during the Cretaceous. Although a sister group relationship is recovered, ancestral state reconstruction using maximum likelihood-based methods reveals two independent origins of photosymbiosis, one at the base of Tridacninae and the other within a symbiotic Fraginae clade.

**Conclusions:**

The newly revealed common ancestry between Tridacninae and Fraginae brings a possibility that certain genetic, metabolic, and/or anatomical exaptations existed in their last common ancestor, which promoted both lineages to independently establish photosymbiosis, possibly in response to the modern expansion of reef habitats.

## Background

Photosymbiotic associations between marine organisms and photosynthetic algae allow each partner to thrive in nutrient-deficient environments and to constitute highly productive ecosystems [1]. This mutualistic relationship has repeatedly evolved in diverse eukaryotic lineages ranging from single-celled foraminiferans to metazoans, including corals, acoels, sacoglossans and bivalves [2]. Although the immediate threat of coral reef symbiont loss (i.e., bleaching) has generated extensive efforts in studying the short-term ecological interactions between coral hosts and symbionts, long-term evolutionary dynamics of photosymbiosis in most marine lineages are poorly understood [3, 4]. Therefore, it is crucial to start investigating the origination and extinction patterns of photosymbiosis in a diversity of organisms and identify tractable study systems, so we can better understand and predict how such systems will respond to long-term environmental change.

Among photosymbiotic host organisms, bivalve mollusks pose an evolutionary dilemma. Compared to other invertebrates that house symbionts in translucent tissues, bivalves possess opaque, protective shells that represent an inherent obstacle to symbiont exposure to sunlight. Therefore, extensive morphological or behavioral adaptations are needed to establish successful photosymbiosis. Nonetheless, at least 17 modern or extinct bivalve lineages are known/suspected to form photosymbioses, and each possess (ed) distinct morphological traits adaptive to photosymbiosis [3]. Within modern taxa, at least seven lineages have been reported to harbor photosynthetic algae [2]. However, obligate and confirmed mutualistic associations are only found in two clades within the bivalve family Cardiidae: the well-known giant clams in the subfamily Tridacninae and the less-studied heart cockles in the subfamily Fraginae [5–8]. Tridacninae has a Late Eocene to Recent fossil record with appearance of symbiont-bearing members from the Late Oligocene. Symbiont-bearing Fraginae appear slightly later in the fossil record, from the Late Miocene to Recent [3]. Comparative studies between these two lineages can reveal important insights into the evolution of photosymbiosis in complex organisms.

Giant clams (Tridacninae) were the first documented photosymbiotic bivalves [5]. A dual feeding strategy coupling photosymbiosis with filter-feeding is hypothesized to have resulted in the enormous size of *Tridacna gigas*, one of the heaviest non-colonial invertebrates known [9]. Giant clams acquire a considerable amount of organic carbon through photosymbiosis [10]. All species in the subfamily possess symbiotic algae from the family Symbiodiniaceae (also found in symbiosis with corals), mostly placed in the genera *Symbiodinium*, *Cladocopium*, and *Durusdinium* [8, 11]. The giant clam harbors extracellular symbionts within a tubular network derived from the digestive system that extends into the mantle tissue [12, 13]. The tubules only develop in the presence of the symbionts [14], indicating the existence of responsive host-symbiont interactions.

**Fig. 1:**
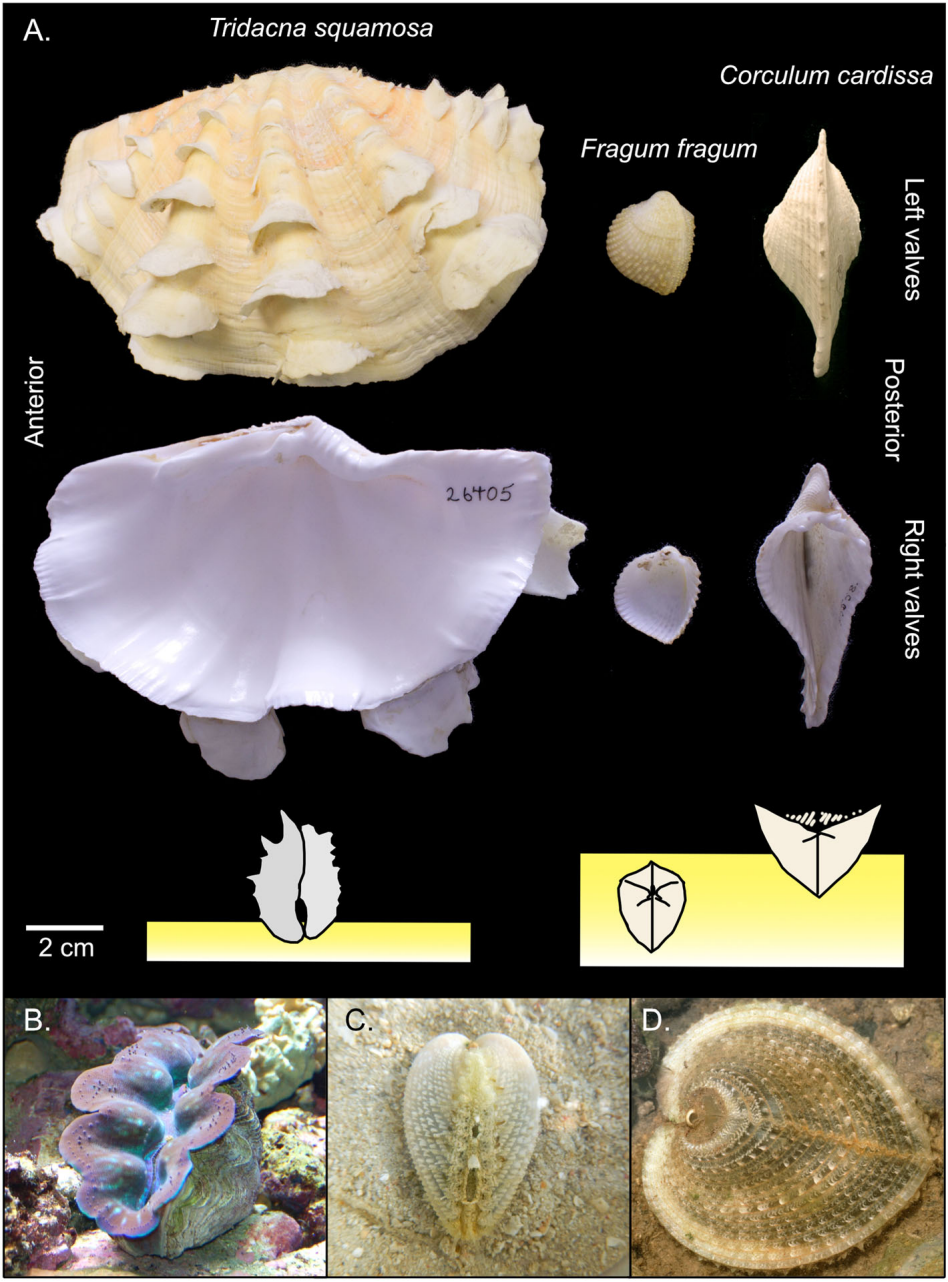
Morphological and ecological comparisons among Tridacninae (*Tridacna squamosa*) and Fraginae (*Fragum fragum*, *Corculum cardissa*) species. **a**. Lateral shell views of the three species and diagrams showing their typical positions in natural habitats. Yellow rectangles represent sediment. Note that *F. fragum* and *C. cardissa* both show different degrees of posterior shell compression. **b**–**d**. Photos of the three species in their natural habitat. *F. fragum* (**c**) was taken out of the sediment when photo was taken. Photo credits: Jingchun Li and Jeff Whitlock (the Online Zoo). Images were processed in Affinity Designer 1.8.4 (Serif Ltd.)

Ecological and morphological adaptations for the symbionts to obtain sufficient sunlight are apparent in Tridacninae. Unlike most species in the family Cardiidae, tridacnines do not bury in sediment. Instead, they are epibenthic (Fig. 1a) with a great expansion of the posterior body, widely gaping shells and a hypertrophied mantle that is exposed to the light (Fig. 1b). The mantle tissues also contain iridophores that scatter photosynthetically productive wavelength to symbionts distributed in the deeper regions [15].

The subfamily Fraginae is a more diverse but less studied group compared to the Tridacninae. It contains more than 50 species, including one symbiotic clade and one non-symbiotic clade, both reciprocally monophyletic [7]. The photosymbiotic species are exclusive to three genera: *Fragum*, *Corculum*, and *Lunulicardia*, comprising at least 23 species [7]. Part or most of their energy source is derived from algal photosynthesis [8]. Symbiont diversity within Fraginae is less explored, but species examined so far harbor algae belonging to Symbiodiniaceae genera *Symbiodinium* and *Cladocopium* [8]. Photosymbiotic fragines host algae within not only the mantle, but also the gill and part of the foot. The symbiont-containing structure is remarkably similar to that documented for giant clams – an extracellular tubular network branching into the symbiont-containing tissues [7, 8, 16].

Despite the similarities between Fraginae and Tridacninae with regards to symbiont identity and symbiont-containing structures, morphological and behavioral adaptations are otherwise quite variable. Photosymbiotic fragines are relatively small sized (~ 1–10 cm) and live in a diverse spectrum of habitats, ranging from shallow reef flats with clear waters to deeper lagoons [7]. Some species are entirely epifaunal (Fig. 1a, d), the rest are shallowly buried in the sediment, as in non-symbiotic cardiids (Fig. 1a). Morphologically, the shells of some species are very similar to those of non-symbiotic fragines and expose symbionts to light via shell gaping (Fig. 1a, c). In contrast, others exhibit highly flattened translucent shells with sophisticated microstructures to enhance light acquisition, referred to as “shell windows” (Fig. 1a, d [17];). Those morphological variations suggest that fragines may depend on photosymbiosis to different degrees, and have evolved diverse solutions to maintain the associations.

Given that the two modern photosymbiotic bivalve groups both arose within the family Cardiidae but use radically different strategies, it is natural to ask whether they inherited photosymbiosis from a common ancestor or independently evolved the associations. If photosymbiosis were an ancestral character, it would imply a single origin with subsequent losses of symbiosis in non-symbiotic fragines and possibly other cardiids. If the two subfamilies acquired photosymbiosis separately, it would suggest that their symbiont-harboring system and other metabolic adaptations are a result of convergent evolution.

In order to address the acquisition of photosymbiosis in bivalves, a phylogenetic framework is required. Currently, the phylogenetic relationships within Tridacninae and Fraginae are relatively well resolved, and both groups are monophyletic [7, 18, 19]. However, the relationship between the two subfamilies remains poorly understood. One of the first cardiid phylogenies, based on a cladistic analysis of shell microstructure and gut morphology, placed Tridacninae and Fraginae as sister groups [20], but later work incorporating more characters resolved the two subfamilies as distantly related lineages within Cardiidae [21]. The first molecular phylogeny constructed to address this issue was based on a single genetic marker (nuclear 18S rRNA), grouping Tridacninae with non-symbiotic cardiids in the subfamilies Trachycardiinae and Laevicardiinae, leaving Fraginae as sister group to the rest [22]. The most recent multi-gene phylogenetic analyses also failed to reliably resolve the positions of Tridacninae and Fraginae [19]. The placement of Tridacninae was not consistent and was recovered as sister group to various subfamilies (e.g., Lymnocardiinae and Cardiinae) in different analyses. The same situation characterizes the placement of Fraginae as its phylogenetic position has shifted with different phylogenetic reconstruction methods. A previous thesis [23] utilizing the same multi-gene dataset, however, did recover a sister relationship between Tridacninae and Fraginae in one Bayesian analysis. Nonetheless, these conflicting results highlight the difficulties in resolving relationships based on three Sanger-based markers, thus hindering our understanding of the evolution of photosymbiosis in bivalves.

The recent development of phylogenomic approaches to bivalve phylogenies [24–26] has demonstrated the possibility of resolving difficult nodes according to previous Sanger-based approaches (e.g., [27]). Therefore, we adopted an RNAseq approach and generated transcriptomic data for Tridacninae, Fraginae and other cardiids with the aim to better understand the evolutionary of photosymbiosis in Cardiidae.

## Results

### Transcriptome assembly and data quality

**Fig. 2:**
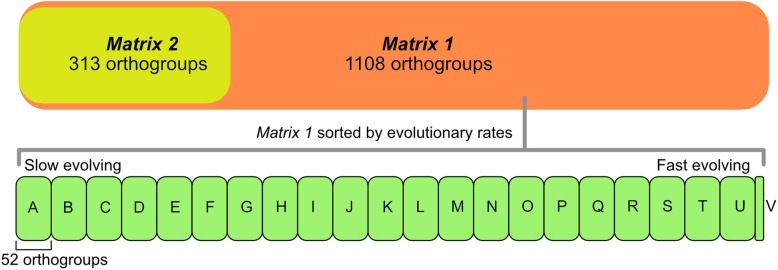
Gene occupancy diagram showing the 25 matrices analyzed in this study. Top: Main matrices of two minimum taxon occupancy thresholds. Orthogoups in Matrix 1 and 2 are shared by at least 50 and 75% of all taxa. Bottom: Matrix 1 was divided into 22 sub matrices based on gene evolution rates from slow (**A**) to fast (V). Each matrix containing 52 orthogroups, except for the last matrix V, which contains 16 orthogroups

The number of used reads, contigs, and other values to assess the quality of the assembled transcriptomes can be found in Table 1. Orthology assessment of this 33-taxon dataset with the OMA stand-alone algorithm recovered 244,752 orthogroups. The two super-matrices (Fig. 2 and Additional file 3: Fig. S1) respectively yielded 1108 (*Matrix 1*: occupancy of > 50%; 280,086 aa) and 313 (*Matrix 2*: occupancy of > 75%; 78,272 aa) orthologs. Levels of compositional heterogeneity were low in most orthogroups (Additional file 4: Fig. S2). The relative composition frequency variability (RCFV) per taxon and per amino acid ranged from 0.0002 to 0.001, representing overall compositional homogeneity throughout all amino acids and taxa included in *Matrix 1*.

**Table 1:** Information of all specimens used in this study. Photosymbiotic taxa are bolded. Abbreviations are as the following. *UCM* Museum of Natural History, University of Colorado Boulder, *FMNH* the Field Museum, *WA* Western Australian Museum, *UMMZ* Museum of Zoology, University of Michigan, *MCZ* Museum of Comparative Zoology, Harvard University, *SRA* NCBI Sequence Read Archive

Species	Locality	Voucher	Tissue	SRA	Contigs	N50	Source
*Fulvia lineonotata*	Guam	UCM48073	Mantle	SRR11252446	243,326	688	De novo
*Americardia media*	USA	FMNH 315293	Muscle	SRR11252445	182,732	527	De novo
***Corculum cardissa***	Australia	WA263	Mantle	SRR11252439	371,151	483	De novo
***Fragum fragum***	Guam	UCM48094	Mantle	SRR11252438	608,501	460	De novo
***Fragum mundum***	Australia	WA265	Whole	SRR11252437	333,455	492	De novo
***Fragum scruposum***	Guam	UCM48090	Mantle	SRR11252436	376,388	508	De novo
***Fragum unedo***	Australia	BivAToL-75 (FMNH)	Muscle	SRR8217860	121,185	393	[26]
***Lunulicardia*sp*.***	Australia	FMNH 317975	Muscle	SRR8217812	244,397	404	[26]
*Microfragum festivum*	Australia	WA261	Whole	SRR11252435	174,309	548	De novo
*Laevicardium serratum*	USA	BivAtoL-57 (FMNH)	Muscle	SRR8217867	162,615	536	[26]
*Laevicardium* sp*.* Curaçao	Curaçao	BivAtoL-456 (FMNH)	Muscle	SRR11252434	196,243	472	De novo
*Acanthocardia tuberculata*	Italy	UMMZ 39326	Mantle	SRR11252433	241,101	554	De novo
*Cerastoderma edule*	England	BivAToL-21 (FMNH)	Muscle	SRR8217858	120,092	594	[26]
*Vasticardium pectiniforme*	Japan	UCM48108	Muscle	SRR8217813	144,372	485	[26]
*Vasticardium compunctum*	Japan	UCM48123	Muscle	SRR11252432	134,451	478	De novo
*Dallocardia muricata*	USA	BivAtoL-454 (FMNH)	Muscle	NA	177,010	368	De novo
*Papyridea lata*	Curaçao	MCZ 383047	Muscle	SRR8217866	267,529	345	[26]
*Trachycardium egmontianum*	USA	FMNH 344567	Muscle	NA	186,788	350	De novo
***Hippopus*sp*.***	Japan	UCM48120	Mantle	SRR11252444	187,464	406	De novo
***Tridacna crocea***	Cultured	UMMZ 304399	Mantle	SRR11252443	251,344	1092	De novo
***Tridacna derasa***	Cultured	UMMZ 304400	Mantle	SRR11252442	245,928	1056	De novo
***Tridacna maxima*2**	Cultured	UMMZ 304398	Mantle	SRR12252441	298,624	956	De novo
***Tridacna maxima*1**	Cultured	NA	Muscle	SRR8217859	176,478	527	[26]
***Tridacna squamosa***	Cultured	UMMZ 304401	Mantle	SRR12252440	245,781	1111	De novo
Outgroup							
*Cardites antiquatus*	Spain	MCZ 379178	Muscle	SRR1560458	113,906	567	De novo
*Chama macerophylla*	Panama	MCZ 381299	Muscle	SRR8217830	165,533	686	De novo
*Corbicula fluminea*	USA	BivAToL-242 (FMNH)	Muscle	SRR1559272	176,007	763	[24]
*Galeomma turtoni*	Spain	MCZ 378975	Muscle	SRR1560274	92,358	548	[24]
*Hiatella arctica*	UK	BivAToL-195 (FMNH)	Muscle	SRR1560281	73,557	576	[24]
*Lyonsia floridana*	USA	BivAToL-248 (FMNH)	Muscle	SRR1560310	92,076	838	[24]
*Mya arenaria*	NA	MCZ 381391	Muscle	SRR1560361	98,870	873	[24]
*Neotrigonia margaritacea*	Australia	MCZ 379092	Muscle	SRR1560432	162,657	549	[24]
*Calyptogena gnifica*	USA	NA	Mantle	SRR8217831	42,445	399	[26]

### Phylogenetic analyses and gene evolution

All 26 analyses (Table 2) recovered strong support for the monophyly of Tridacninae, Trachycardiinae, Laevicardiinae, Cardiinae, Lymnocardiinae, photosymbiotic Fraginae, and non-symbiotic Fraginae. Monophyly of Fraginae (including both symbiotic and non-symbiotic clades) was supported in 63% of the analyses. The clade composed of Trachycardiinae, Laevicardiinae, and Cardiinae was supported in all analyses. The placement of Lymmnocardiinae was less certain, although 60% of the analyses recovered it as the sister group to all other Cardiidae.

**Table 2:** A summary of phylogenetic analyses conducted in this study

Gene Matrix	Analysis	Model
*1,2, A-V*	PhyML	PCMA
*1*	RAxML-Dayhoff	Multigamma GTR
*2*	PhyloBayes	CAT-GTR
Individual genes	RAxML	PROTGAMMALG4X

**Fig. 3:**
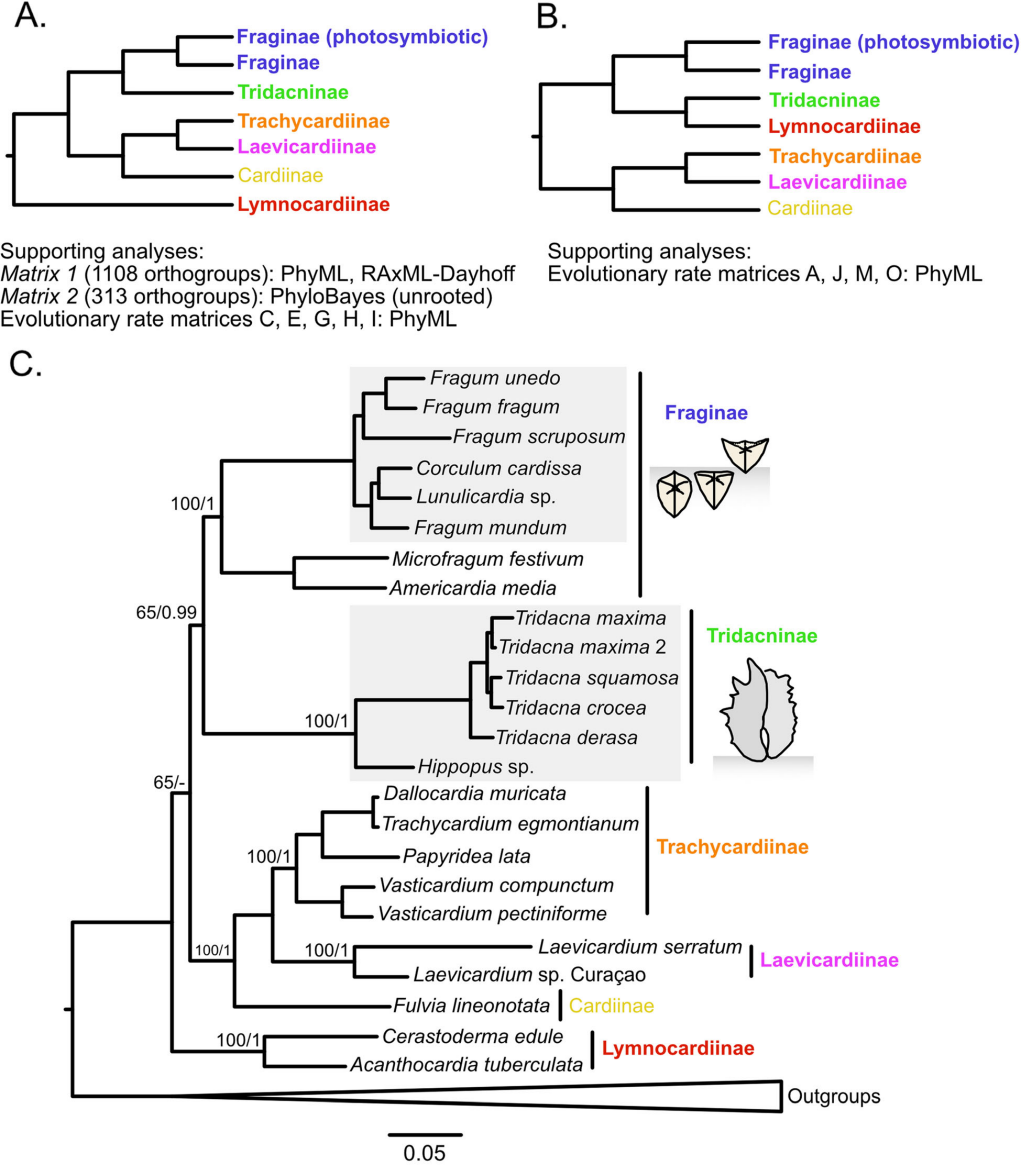
**a**-**b**. The two best supported topologies obtained from the analyses of the two main matrices and 22 submatrices. Cardiid subfamilies are indicated by different colors. Supporting matrices and corresponding analytical methods are listed under each topology. **c**. Phylogenetic results based on maximum likelihood analysis (PhyML-PCMA) of Matrix 1 and Bayesian analysis (PhyloBayes) of Matrix 2. This is also the topology supported by the most analyses. Node labels represent bootstrap supports / posterior probabilities of each subfamily and the backbone. Photosymbiotic clades are shaded in grey. Shell position of Fraginae and Tridacninae species in their natural habitats is shown in the two diagrams

The most supported overall topology by different analyses (Fig. 3a) is consistent with the result obtained from the maximum likelihood (ML) analysis on *Matrix 1* (Fig. 3c), which revealed a sister group relationship between Tridacninae and Fraginae, although with moderate bootstrap support (65/100). After Dayhoff recoding of *Matrix 1*, the same topology was obtained, as for the PhyloBayes analysis of *Matrix 2*, although the two independent chains did not fully converge. The conflict was mostly driven by the position of Lymnocardiinae as the sister group to all other cardiids. The posterior probability for the Tridacninae and Fraginae sister group relationship is 0.99 and does not show conflict between the two chains. Analyses of submatrices C, E, and G-I also recovered this common topology. The second most common topology is largely consistent with the first one, except that Lymnocardinae is placed as the sister group of Tridacninae (Fig. 3b). Other matrices supporting this topology include the slowest evolving submatrix A, and three relatively fast evolving ones (J, M, O).

Besides the most supported topologies, an additional 11 topologies were supported by only one or two matrices each. In particular, ML analysis on *Matrix 2* placed Fraginae as the sister group to a clade composed of (((Trachycardiinae, Laevicardiinae), Cardiinae), Lymnocardiinae), and recovered Tridacninae as the sister group to all other Cardiidae. However, the bootstrap support values for the backbone topology are low (< 60).

The supernetwork analyses on the two main matrices showed relatively long branches leading to Tridacninae, photosymbiotic Fraginae, and non-photosymbiotic Fraginae, respectively (Additional file 5: Fig. S3), a result consistent with the strong support for these nodes obtained from the phylogenetic analyses. However, substantial reticulation was observed at the node leading to these three clades, indicating strong gene conflict in resolving their relationships. This is likely the reason for the low bootstrap supports obtained in the ML analyses.

BLAST results for the fastest 10% and slowest 10% genes are shown in Additional files 1 and 2: Table S1 and S2. The slow-evolving genes are composed of a higher proportion of nuclear ribosomal genes compared to the fast evolving ones (13% vs. 0%). The fast-evolving genes have a higher number of mitochondrial genes compared to the conserved genes (23% vs. 1%). This pattern is in line with what is currently known about the molecular evolution rates of these gene groups.

The coral V-type H + -ATPase (VHA, photosymbiosis-related gene) BLAST searches recovered VHA sequences from eight Tridacninae and Fraginae species. Tridacninae and Fraginae VHA amino acid sequences are mostly identical, except for *Fragum fragum,* which has three (out of 137) amino acid substitutions. Besides *F. fragum*, there are no sequence differences between the photosymbiotic and non-symbiotic taxa (Additional file 6: Fig. S4). The coral VHA differs from bivalve sequences by 6–11 amino acid substitutions. Both coral and bivalve sequences share ~ 85% similarity with the Symbiodiniaceae VHA. All animal VHA genes formed a monophyletic group with 100% bootstrap support.

### Fossil calibration and ancestral state reconstruction

**Fig. 4:**
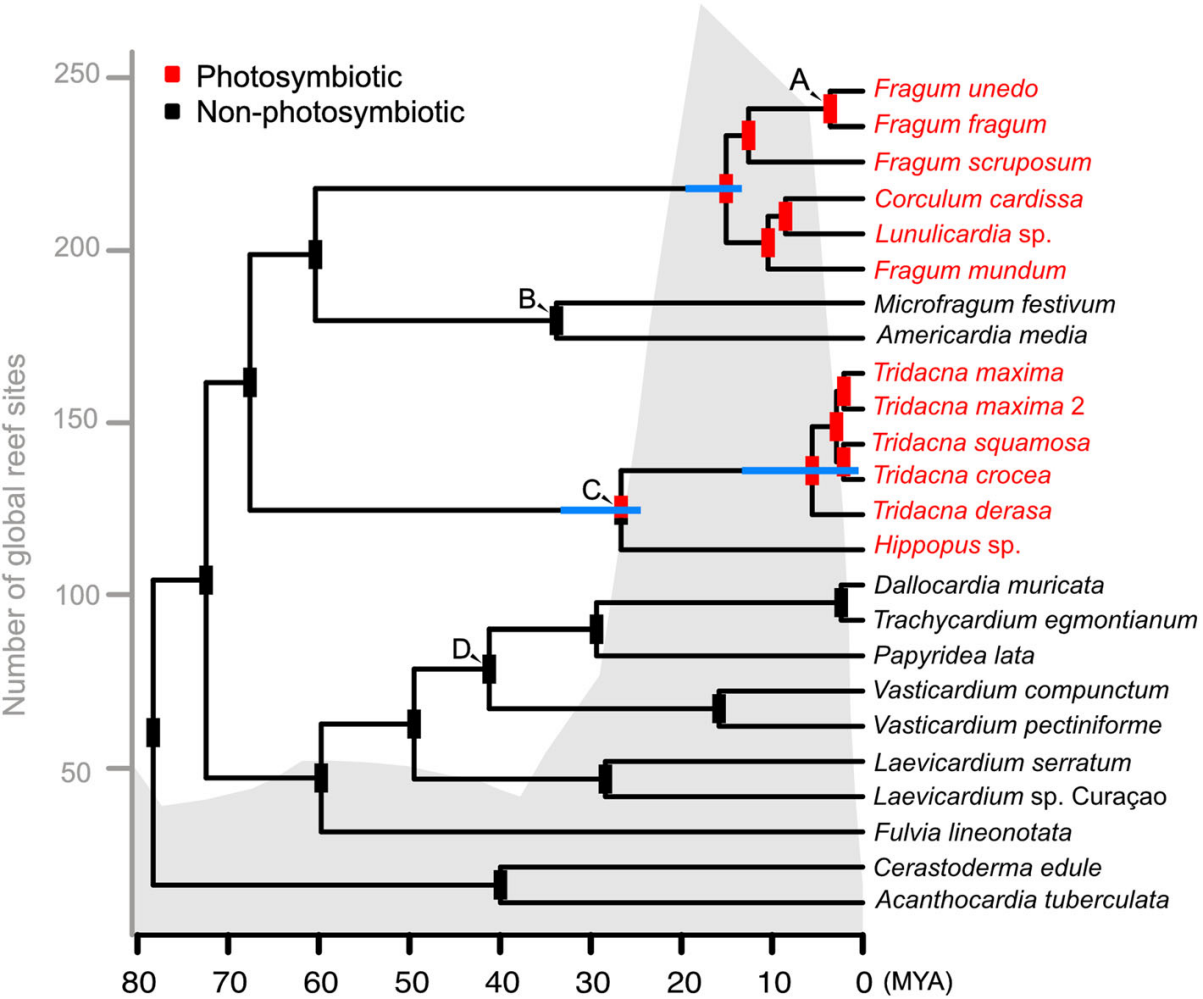
Fossil calibrated phylogeny and ancestral state reconstruction based on the most supported topology. Letters (**a**–**d**) at nodes indicate calibration points. Blue bars at nodes represent standard deviation of age estimation. Red and black bars at nodes represent probabilities of the common ancestor being photosymbiotic (red) or non-photosymbiotic (black). The grey shading in the background corresponds to number of global reef sites through time (following [28])

The cardiid dating estimates based on the most common topology are shown in Fig. 4. Tridacninae and Fraginae diverged in the Late Cretaceous. Soon after, the symbiotic and non-symbiotic Fraginae lineages also separated. Those long branches eventually lead to relatively recent and rapid diversification of the photosymbiotic crown groups. The diversification of photosymbiotic fragines is dated at around 15 Ma (SD = 3.1). The diversification of crown Tridacninae is ~ 27 Ma (SD = 4.4), and the radiation of the genus *Tridacna* dates back to ~ 6 Mya (SD = 6.3). Age estimates of the other non-focal subfamilies, including Trachycardiinae, Laevicardiinae, Cardiinae, and Lymnocardiinae, are largely within the range of Herrera et al. 2015 [19].

Ancestral state reconstructions using the Binary State Speciation and Extinction (BiSSE) and mk2 models yield congruent results (Fig. 4). Both identified two independent origins of photosymbioses in Cardiidae, one within Tridacninae and the other within the photosymbiotic Fraginae. Comparison with reef abundance through time shows that the onset of Tridacninae photosymbiosis corresponds to the beginning of a rapid global reef expansion, while radiations of the genus *Tridacna* and photosymbiotic fragines both occurred within peak reef abundance.

## Discussion

### Cardiid phylogeny

This is the first phylogenetic study using transcriptomic data that aims to resolve the relationships between the two cardiid subfamilies which contain photosymbiotic taxa. The best corroborated hypothesis supports a sister relationship between Tridacninae and Fraginae, both recovered as monophyletic (Fig. 3). Other than the position of Tridacninae, the placement of all other cardiid subfamilies is consistent with the most recent multi-gene Cardiidae phylogeny comprising 110 species [19].

Our analyses highlighted the pervasive challenge to reconstruct a well-resolved and highly supported cardiid phylogeny. The fundamental reconstruction difficulty stems from the diversification process of Tridacninae, photosymbiotic, and non-symbiotic Fraginae. Our results show that the branches leading to the three crown groups are long and subtended by very short internodes, indicating the divergence among these groups was rapid. These ancient but fast diversification events are inherently difficult to resolve [29]. Some of the major problems include: not enough data to resolve the nodes; molecular data not variable enough at the appropriate level; strong conflicts among genes; and inadequate substitution models [29]. We discuss these concerns in the following paragraphs.

Firstly, current studies on cardiid phylogeny may suffer from some level of data limitation. For example, although Herrera et al. 2015 [19] had excellent taxon sampling through Cardiidae, the gene coverage was low. In the present study, taxon sampling was limited due to the need to obtain high quality transcriptomes, which requires freshly collected specimens. These constraints could be overcome by incorporating DNA-based phylogenomic approaches (e.g., RADseq, exome capture, etc.) using well-preserved museum specimens.

In addition, some of the data may lack the appropriate level of resolution. For example, the ML Analysis on *Matrix 2* resulted in poorly resolved topologies with low bootstrap support values. This observation where large, albeit more incomplete matrices provided better resolution than small and more complete matrices is not unique to our dataset (e.g., [24, 30]). It is likely influenced by gene choice in the more complete matrix [30]. If a gene is present in more taxa, it is likely to be relatively conserved; hence lacking enough genetic variation to resolve rapid divergence, as seen in some of our individual gene trees. These very conserved/slow-evolving genes might be over-represented in a smaller matrix, but contribute minimally to any phylogenetic resolution.

Gene conflicts are also apparent in our dataset based on the supernetwork and submatrix analyses, and in part may be explained by incomplete lineage sorting at these rapid-divergence nodes. In addition, genes with different rates of evolution gave rise to divergent topologies. Some faster evolving genes (e.g., Matrices Q-V) produced clearly problematic topologies, such as placing photosymbiotic Fraginae as the sister clade to all other cardiids. These rapidly evolving genes might be subject to more genetic saturation and could be under the influence of strong selection, all of which hinder their ability to resolve deep phylogenetic nodes and conflict with other more informative genes.

Lastly, sequence compositional heterogeneity is known to be especially problematic for inferring short internal nodes [31]. However, based on the low level of composition heterogeneity in our dataset and the Dayoff recoding analysis, it is unlikely to produce significant bias in the results.

In sum, we have found that overly conservative genes and fast evolving genes do not provide informative resolution to the nodes of interest, a phenomenon observed in other taxonomic groups [32]. These genes generate conflicting topologies and could be responsible for the observed low bootstrap values. On the contrary, ML analyses on the large matrix, moderately evolved genes, as well as the Bayesian analysis, all support a sister relationship between Tridacninae and Fraginae.

### Implications on photosymbiosis evolution

Our analyses support two independent origins of photosymbiosis in Cardiidae. This result should be robust to phylogenetic uncertainties, because a non-sister relationship between Tridacninae and Fraginae will only reinforce the independent evolution scenario. Our ancestral state reconstruction was model-based; an alternative parsimony-based analyses would deem “two independent origins” or “one origin and one loss (in the non-symbiotic Fraginae clade)” equally likely. However, the latter scenario is not well supported because fossil record indicates that visible shell adaptations in both Tridacninae and Fraginae appeared after the Late Oligocene [3, 33]. If the common ancestor of the two subfamilies is photosymbiotic, it would imply that photosymbiosis was established in late Cretaceous but persisted more than 30 Ma without any apparent shell adaptations. It is much more probable that there are two separate origins of photosymbiosis with shell adaptations evolved shortly after.

The repeated evolution of bivalve photosymbiosis suggests its adaptive advantage. The relatively rapid crown group diversification, coupled with morphological responses [3], is consistent with criteria for adaptive radiation - the generation of new species exhibiting pronounced morphological divergence over relatively short timeframes, typically in response to new environmental conditions [34]. In photosymbiotic cardiids, the species number is modest compared to other documented examples. However, this might be due to the lack of systematics research in these groups, as more studies have started to reveal their hidden diversities (e.g., [35]).

As for most cardiid lineages, Tridacninae and Fraginae have inferred ancestral distributions that span the Indo-Pacific; Tridacninae has a wider ancestral range, reaching the western temperate northern Pacific [19]. Given that the origin of photosymbiosis in both subfamilies overlaps with the expansion of modern reefs, it is likely that the formation of shallow marine habitat in the Indo-Pacific is a key environmental driver for bivalve photosymbiotic adaptation. This is further supported by the fact that photosymbiotic fragines have a sister non-symbiotic lineage that still inhabits deep sandy bottoms [7], possibly representing the ancestral ecology of these bivalves before they shifted to shallower habitat. Kiessling 2009 [28] proposed a fundamental question regarding the formation of reef biodiversity – Have reef taxa originated within reefs or have they evolved somewhere else then moved into reef habitats? Our results indicate that at least for photosymbiotic bivalves, they have likely originated and diversified within the reef habitats. More biogeographical, palaeontological and phylogenetic data are certainly needed to further corroborate this point.

Both Tridacninae and photosymbiotic Fraginae are thought to exhibit phenotypic adaptations to benefit the symbionts, making some species’ shell and mantle morphologies drastically different from typical cardiids (Fig. 1). What is striking, and little mentioned until now, is the divergent morphological trends in photosymbiotic fragines contrasted with the uniform morphological trends in Tridacninae, essentially two different responses to expose symbionts to optimal irradiance in sister lineages. Previous studies of adaptive radiations have well highlighted examples of divergent morphological responses to newly available niches (e.g., [36]), as seen in fragines. But uniform morphological responses, as seen in tridacnines, are less documented. Both strategies have advantages. While divergent morphologies provide highly specialized adaptations to different fine-scale niches, an uniform/static morphology may enable the lineages to become broadly adapted generalists to mediate environmental fluctuations [37]. The different morphological evolution patterns in photosymbiotic cardiid suggests that in the acquisition of a key innovation, historical morphological contingencies (e.g., opaque heavy shell, unexposed tissues, infaunal habit) and common ancestry do not predict the directionality of morphological evolution, not even for sister lineages that use the same ecological strategy to adapt to similar habitats.

Despite the versatile shell and mantle morphologies in Fraginae and Tridacninae, their symbiont-containing tubular system share striking similarities. Both stem from the digestive system of the hosts and form tertiary tubular networks [13, 16]. The only other similar molluscan structures are found in some marine gastropods who temporarily maintain algae or chloroplasts in their tissues [38]. The development of giant clam tubules are only triggered by the presence of symbionts [14], indicating the acquisition of photosymbiosis is a highly interactive process between hosts and symbionts.

It has long been hypothesized that different photosymbiotic bivalves express homologous genes to build the tubular system, in response to similar symbiont signals [16]. The newly-found sister relationship between Tridacninae and Fraginae lend further support to this theory, suggesting that genes homologous to the tubular-formation ones are ancestral to the two lineages. It is even possible that the genes are ancestral to bivalve and gastropods, as the latter can form similar anatomical structures. Given that the different mollusk lineages evolved photosymbiosis independently, it is likely that the gene/anatomical level similarities are generated from parallel evolution. That is, each lineage independently coopted similar genetic mechanisms for generating symbiont-containing structures. Molecular level parallel evolution has been shown in photosymbiotic systems. For example, both corals and giant clams repurpose the expression of the vacuolar H + -ATPase gene (VHA) to facilitate their carbon concentrating process and promote algal photosynthesis, even though the two lineages are very distantly related [39, 40]. Our analyses further support that VHA is a conserved gene, ancestral to bivalves, corals, and other animals. Photosymbiotic bivalves do not possess any “special” version of VHA; the amino acid sequence is the same as the ones found in non-symbiotic taxa. It is more likely that the adaptation to photosymbiosis is realized at the regulatory/expression level, where VHA is highly expressed in tissues that harbor symbionts. In contrast with the relatively labile shell and mantle adaptations, it is possible that the evolution of host metabolomic and developmental adaptations are more constrained genetically, resulting in similar mechanisms in diverse animal groups. More in-depth studies on the molecular mechanisms behind photosymbiosis are needed to gain better insights.

To further investigate macroevolution of bivalve photosymbiosis, a better understanding of potential photosymbiotic fossil taxa is essential. However, they are challenging to identify in the fossil record based on shell morphology alone [2]. Although photosymbiotic bivalves possess a suite of morphological traits to enhance light exposure, almost all characters used to support photosymbiotic condition are found in modern non-photosymbiotic bivalves. Examples include permanent shell gaping (Family Limidae, Galeommatidae), enlarged mantle (Galeommatidae), or transparent shells (Placunidae, Pinnidae). The only photosymbiotic-exclusive shell character is the shell “window” microstructure [17], but many photosymbiotic bivalve species do not possess it, and similar features have not been found in fossil taxa. Similarly, other identifiable photosymbiotic ecological traits (shallow water distribution, fast growth, reclining habits) are not unique to photosymbiotic bivalves either [2]. Therefore, alternative methods need to be developed (e.g. better isotopic metrics, metabolite signatures) if we wish to fully understand the evolutionary dynamics of bivalve photosymbiosis.

Lastly, further characterization of host-symbiont diversity and interactions are essential for developing a full picture of animal-algal photosymbiosis. A comprehensive documentation of host-symbiont biodiversity can provide insights into photosymbiotic mechanisms in diverse habitats and with different organismal complexities. For example, Li et al. 2018 [8] demonstrated that fragine species at varying water depths have different dependencies on algal photosynthesis, with deeper host species utilizing less photosynthetically derived carbon and exhibiting fewer shell modifications. Therefore, it is highly likely that host-symbiont interactions and reliance vary greatly among host-symbiont pairs, depending on symbiont types, as well as the degree of host adaptations. Li et al. 2018 [8] summarized known symbiont diversity in giant clams and showed that several fragine species harbor symbionts from the genus *Cladocopium*, which also occupies cnidarians, giant clams and foraminifera. However, this by no means captured the full spectrum of potential symbiont diversity in photosymbiotic bivalves, especially when new host species are discovered regularly (e.g., [35]). In most photosymbiotic bivalve systems, the roles symbionts play in host ontogeny, reproduction, response to environmental fluctuation, etc. are unexplored, all of which require our continued effort to identify and characterize existing photosymbiotic diversity.

## Conclusions

This study revealed that two closely related bivalve subfamilies independently evolved photosymbiosis, further demonstrating the prevalence of photosymbiosis in marine ecosystems. Selection pressure for photosymbioses is high in oligotrophic environments, because the associations provide immediate metabolic benefits to the partners [41]. In some parts of the ocean, the diversity and abundance of photosymbiotic plankton hosts are substantially higher than that of phototrophic protists [42]. However, despite the ecological and evolutionary importance of photosymbioses, our knowledge of their origin, diversity, environmental impact, and genetic mechanisms remains rudimentary [41]. The evolution of photosymbioses has been hypothesized to drive rapid diversification, have significant impacts on the community composition, and influence productivity and biogeochemical cycling of the ecosystem [43]. Most of these hypotheses have not been formally tested or have only been tested on a small number of lineages. Therefore, it is now time to move beyond a few model groups and start to comparatively address evolutionary patterns, genomic adaptations, and geological impacts of diverse photosymbiotic systems.

## Methods

### RNA extraction, Transcriptome sequencing, and assembly

We obtained transcriptomes of 24 Cardiidae specimens and 9 bivalve outgroups. Information about the sampled specimens can be found in Table 1 and in the MCZ online collections database (http://mczbase.mcz.harvard.edu).

Bivalve tissues were collected fresh and flash frozen in liquid nitrogen or preserved in RNAlater® (Life Technologies) and stored at − 80 °C. Total RNA from bivalve mantle and foot tissues was extracted and purified as described in [26]. mRNA quality was assessed with a picoRNA assay in an Agilent 2100 Bioanalyzer (Agilent Technologies) and quantity was measured in a Qubit fluorometer (Life Technologies). 28S rRNA in mollusk samples breaks down into two segments of comparable sizes to 18S rRNA during Bioanalyser quality assessment, resulting in non-meaningful RIN scores [44–46]. Therefore, we used the RNA electropherogram itself to validate the RNA integrity. When a significant single peak (composed of 18S and the broken 28S) was observed, it indicated good RNA integrity.

The cDNA libraries were constructed with an Apollo 324 NGS Library Prep System (TakaraBio) as described in [26]. cDNA library concentrations, quality and sizes were assessed as in [26] before being sequenced on an Illumina HiSeq 2500 platform with paired-end reads of 150 bp at the FAS Center for Systems Biology at Harvard University. Tridacninae samples were sequenced at the Advanced Genomics Core, University of Michigan.

Demultiplexed sequencing results were retrieved in FASTQ format. Each sample was processed in the following steps. Trimgalore 0.3.3 [47] was used to quality filter the data and trim adapters (all reads with an average Phred score lower than 30 and shorter than 25 bp, were discarded). Ribosomal RNA (rRNA) was filtered out using Bowtie 2.0.0 [48] by building a bowtie index using all known mollusk rRNA sequences downloaded from GenBank. Some of the bivalve tissues (mantle) used for RNA extraction contain symbionts, whose expressed genes could interfere with the transcriptome assembly. Therefore, Symbiodiniaceae reads were filtered out of photosymbiotic bivalve sequences (Tridacninae and Fraginae) by aligning reads to the transcriptomes of Symbiodiniaceae strains from multiple genera [49–51]. All reads that did not align with the rRNA or Symbiodiniaceae indices were stored in FASTA format as single files and used in our downstream analyses.

Detailed methods for de novo transcriptome assembly, filtration and translation can be found in [26]. In brief, assemblies were conducted with Trinity r2014-04-13 [52, 53], filtration of redundant reads with CD-HIT version 4.6 [54], translation with TransDecoder [53] and isoform filtration with a custom Python script. All reads generated for this study are deposited in the National Center for Biotechnology Information Sequence Read Archive (NCBI-SRA; Table 1) and all assembled transcriptomes can be found in the online Harvard databse: https://dataverse.harvard.edu/dataverse/cardiid_transcriptome.

### Orthology assignment, gene matrix construction

Orthologous genes across all 33 taxa were identified for downstream phylogenetic analyses using OMA stand-alone v0.99z.3 [55, 56]. Contrary to standard bidirectional best-hit approaches, the OMA algorithm uses evolutionary distances instead of scores, considers distance inference uncertainty and differential gene losses, and includes many-to-many orthologous relations; making it more powerful in identifying true orthologs [57]. The ortholog matrix is constructed from all-against-all Smith–Waterman protein alignments. The algorithm then identifies stable ortholog pairs, verifies them, and checks against potential paralogous genes before clustering cliques of stable pairs as groups of orthologs.

For each of the 244,752 unique orthogroups predicted by OMA, amino acid sequences from all available taxa were aligned using MUSCLE version 3.6 [58]. Positions showing poor alignment scores were identified and discarded using a probabilistic character masking approach with ZORRO [59] (selected parameters are detailed in [26]).

Two initial gene matrices were generated by selecting orthogroups based on minimum taxon occupancy thresholds ([60]; see Fig. 2 and Additional file 3: Fig. S1). *Matrix 1*, with a minimum gene occupancy of 50% (i.e., each gene is present in at least 50% of the taxa), is composed of OMA orthogroups shared by 17 or more taxa; and *Matrix 2* includes orthogroups shared by 25 or more taxa (gene occupancy > 75%). Orthogroups for the two matrices were then concatenated using Phyutility 2.6 [61]. All data were treated as amino acids and the two matrices do not contain any contaminant sequences from the Symbiodiniaceae symbionts. In addition to filtering out any symbiont genes during the transcriptome assembly process, no Tridacninae or Fraginae exclusive genes (i.e., possibly from algal symbionts) were used for down stream analyses. All genes used in down-stream analyses were strictly shared among symbiotic and non-symbiotic cardiid taxa. 

### Phylogenetic analyses, compositional heterogeneity assessment, and gene function assessment

A summary of all analyses is shown in Table 2. The two main matrices, *Matrices 1* and *2*, were analyzed using maximum likelihood (ML) inferences conducted by PhyML-PCMA with 10 principal components [62] using Subtree Pruning and Regrafting (SPR) and three initial random starting trees with a random seed. One hundred bootstrap replicates were conducted for *Matrices* 2 using PhyML-PCMA. Bootstrap replicates for *Matrix 1* were computed with RAxML 7.7.5 with the fast bootstrap algorithm [63], because of its large size and the intense computation required by PhyML-PCMA.

*Matrix 2* (75% minimum gene occupancy, 313 orthogroups) was also analyzed using a Bayesian inference with PhyloBayes MPI v.1.4e [64]. The heterogeneous CAT-GTR model of evolution [65] was used, accounting for potential site-specific amino acid preferences. Other settings were kept as default. Three independent Markov chain Monte Carlo (MCMC) runs were conducted for 7496–11,852 cycles and their convergence was evaluated with the bpcomp and tracecomp commands.

BaCoCa v.1.1r was used to estimate relative composition frequency variability (RCFV) in *Matrix 1*. RCFV is a measure of the absolute deviation from the mean for each amino acid and for each taxon summed up over all amino acids and all taxa [66]. High values of compositional heterogeneity may negatively impact phylogenetic inferences [67]. RCFV values were plotted in a heatmap using the R package gplots. To assess the impact of compositional heterogeneity, Dayhoff recoding was employed on *Matrix 1*,as described in [26]. This recoding should minimize effects of compositional heterogeneity as well as of long-branch attraction [68]. The recoded matrix was analyzed with RAxML 7.7.5 using multigamma GTR (−m MULTIGAMMA -K GTR).

*Matrices 1* and *2* showed some inconsistent tree topologies across analyses, we thus tested them for putative gene incongruence by generating individual gene trees for each orthogroup included in each of the three main matrices with RAxML 7.7.5 and the PROTGAMMALG4X substitution model [69]. The choice of the PROTGAMMALG4X model was made based on past experience with bivalve transcriptomic data analyses [24–26], where this model of amino acid substitution revealed to consistently be the best fitted for this kind of data. All individual best-scoring trees were concatenated per matrix, intergene conflict was analyzed with SuperQ v1.1 [70], as described in [26] and the super-networks were visualized with SplitsTree v.4.13.1 [71].

Because the individual gene tree super-networks revealed gene conflict at specific nodes (see results for details), we further explored the effects of molecular evolution rate heterotachy on tree topology. All 1108 orthogroups from Matrix 1 were sorted based on their evolutionary rate, as detailed in [26]. The sorted orthogroups were then divided into 22 matrices (Matrices A to V, Fig. 2), each containing 52 sorted loci, except for the last one which contains 16 loci. The first matrix contains the 52 slowest evolving genes (most conserved) and the last contains the 16 fastest evolving genes (least conserved). Each matrix was then analyzed using PhyML-PCMA as described above. In addition, we investigated gene functions of the 10% slowest (matrices A and B) and 10% fastest (matrices T-V) evolving genes. The amino acid sequences were queried against the SWISS-PROT protein sequence database [72] using the NCBI BLAST server [73].

We also assessed the evolution of one particular gene that is known to promote photosymbiosis in *Tridacna* [39] and corals [40], the V-type H + -ATPase (VHA). We identified VHA genes from our Tridacninae and Fraginae species by running a BLAST search using the scleractinian coral (*Acropora yongei*) VHA amino acid sequence reported in [40]. Any bivalve sequences that share a more than 90% identity with the coral VHA were considered bivalve VHA. The bivalve sequences were then aligned with coral and Symbiodiniaceae [40] VHA sequences, as well as Genbank sequences from a mosquito (AAR13789), a fish (BAC75967) and an alveolate protist (XP_002782779). A maximum likelihood phylogeny with 100 bootstraps was constructed using PhyML as described above.

### Fossil calibration and ancestral state reconstruction

A fossil-calibrated phylogeny was generated based on the best-supported topology from multiple analyses (see results and discussion for more details). We applied a penalized likelihood method [74] using a truncated Newton optimization algorithm implemented in r8s 1.81 [75]. This method was chosen to avoid computational limitations imposed by analyzing big datasets in a Bayesian framework, especially given that the two approaches tend to yield similar divergence time estimates (e.g., [76]). The *Matrix 1* ML phylogeny (outgroup removed) was used as the input tree for r8s. Fossil dates representing the earliest occurrences of well-defined taxa/genera were used to constrain the minimal node ages of four groups: *Fragum fragum, Americardia media, Tridacna*, and Vasticardium (Table 3, [77–80]). A cross-validation analysis [62] was performed on the ML tree with smoothing parameters varying from 1 to 1* 10^5^ to determine the optimal parameter. To account for branch length and topological uncertainties, we repeated the analyses using 100 RAxML bootstrap trees obtained from previous phylogenetic analyses on *Matrix 1*. Node age statistics from the 100 analyses were summarized using the *profile* command in r8s.

**Table 3:** Fossil records used for calibrating the cardiid phylogeny

Node	Taxa	Min. node age (MYA)	Source
A	*Fragum fragum*	3.6	[77]
B	*Americardia media*	7.2	[78]
C	*Tridacna*	23	[79]
D	*Vasticardium*	41.2	[80]

To explore the origin of photosymbiosis in Cardiidae, ancestral state reconstructions were performed on the fossil calibrated phylogeny. A maximum likelihood reconstruction was conducted using the Binary State Speciation and Extinction (BiSSE) model [81] on the calibrated *Matrix 1* ML phylogeny. This method was chosen because it is the only method that accommodates incomplete taxon sampling, not because we pre-assumed photosymbiotic and non-symbiotic lineages exhibit different diversification rates. Photosymbiotic and non-photosymbiotic ecologies were coded as discrete characters for each taxon included in the ingroup. Sampling fraction for photosymbiotic (33%) and all non-symbiotic cardiids (6%) were calculated based on the most recent taxon records from the World Register of Marine Species (WoRMS). Given the known caveats of the BiSSE model [82], we also conducted a reconstruction using the mk2 model [83], which does not assume trait-based diversification rates, although the analysis does not take missing taxa into consideration. Both analyses were conducted using the “diversetree” package [84] in R 3.4.3.

Lastly, we compared the timing of bivalve photosymbiotic evolution to the abundance of global reef habitats through time, to assess the importance of habitat availability. The total number of global reef sites for the past 90 million years (following [28]) were overlaid on the fossil calibrated cardiid phylogeny for visual comparison.

## Supplementary information

**Additional file 1 : Table S1.** BLAST search results of the 10% fastest-evolving genes (matrices A, B) used in the phylogenetic reconstruction.

**Additional file 2 : Table S2.** BLAST search results of the 10% slowest-evolving genes (matrices T-V) used in the phylogenetic reconstruction.

**Additional file 3 : Figure S1.** Gene occupancy representation per species for matrices 1 and 2. A white cell indicates a gene that was not sampled. Taxa are sorted from the highest (top) to lowest (bottom) gene representation.

**Additional file 4 : Figure S2**. A heat map of relative composition frequency variability (RCFV) values for all orthogroups in Matrix 1. Species name are coded as in Additional file 3: Figure S1.

**Additional file 5 : Figure S3.** Gene super-network for matrices 1 and 2. Species name are coded as in Additional file 3: Figure S1.

**Additional file 6 : Figure S4.** Maximum likelihood phylogeny of the VHA genes from Fraginae, Tridacninae, the coral *Acropora yongei*, Symbiodiniaceae, and other eukaryotes. Non-photosymbiotic bivalve taxa are labeled red. The bootstrap value for the animal VHA clade is shown above the branch.

## Data Availability

All reads generated for this study are deposited in the NCBI-SRA repository. All transcriptome accession and museum catalogue numbers are listed in Table 1. Fully assembled transcriptomes and phylogenies are deposited in Harvard dataverse (https://dataverse.harvard.edu/dataverse/cardiid_transcriptome).

## Abbreviations

BiSSE: Binary State Speciation and Extinction
MCMC: Markov chain Monte Carlo
ML: Maximum likelihood
RCFV: Relative composition frequency variability
SPR: Subtree pruning and regrafting
VHA: V-type H + -ATPase
WoRMS: World Register of Marine Species
