# Supplementary figures and images for: Shedding light: a phylotranscriptomic perspective illuminates the origin of photosymbiosis in marine bivalves
Source: BMC Evol Biol. 2020 May 1;20:50. doi: 10.1186/s12862-020-01614-7 (PMC7195748; doi:10.1186/s12862-020-01614-7)

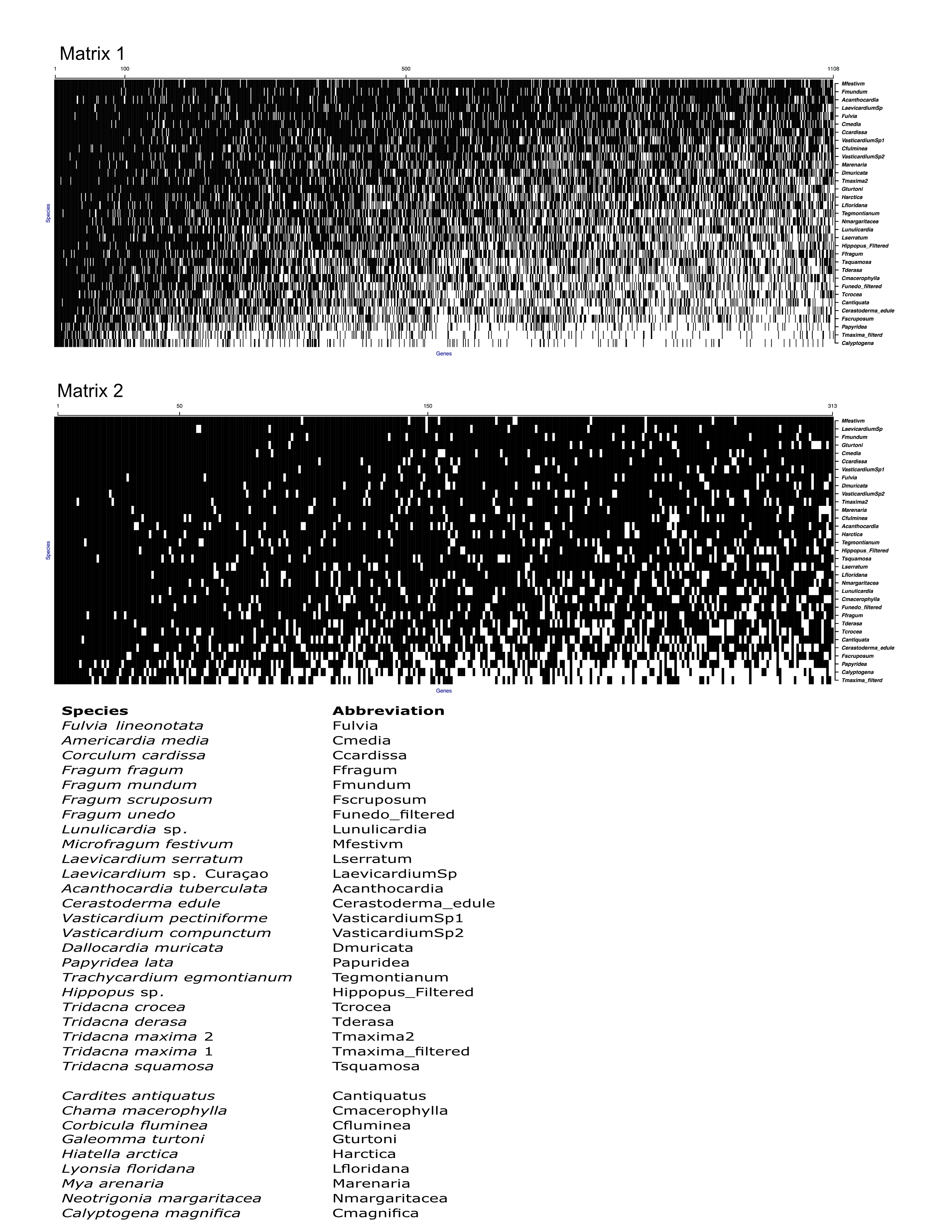

Supplement: Supplementary file 3 — Additional file 3 : Figure S1. Gene occupancy representation per species for matrices 1 and 2. A white cell indicates a gene that was not sampled. Taxa are sorted from the highest (top) to lowest (bottom) gene representation. [file 12862_2020_1614_MOESM3_ESM.jpg]

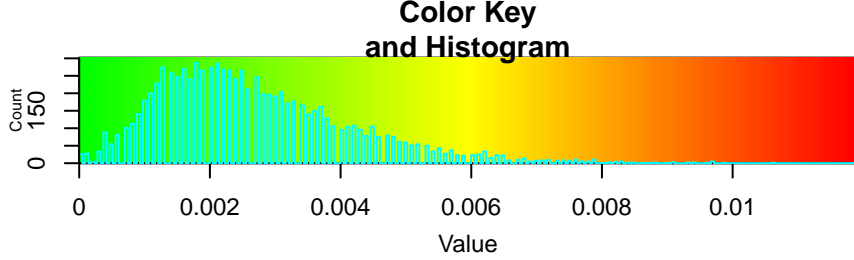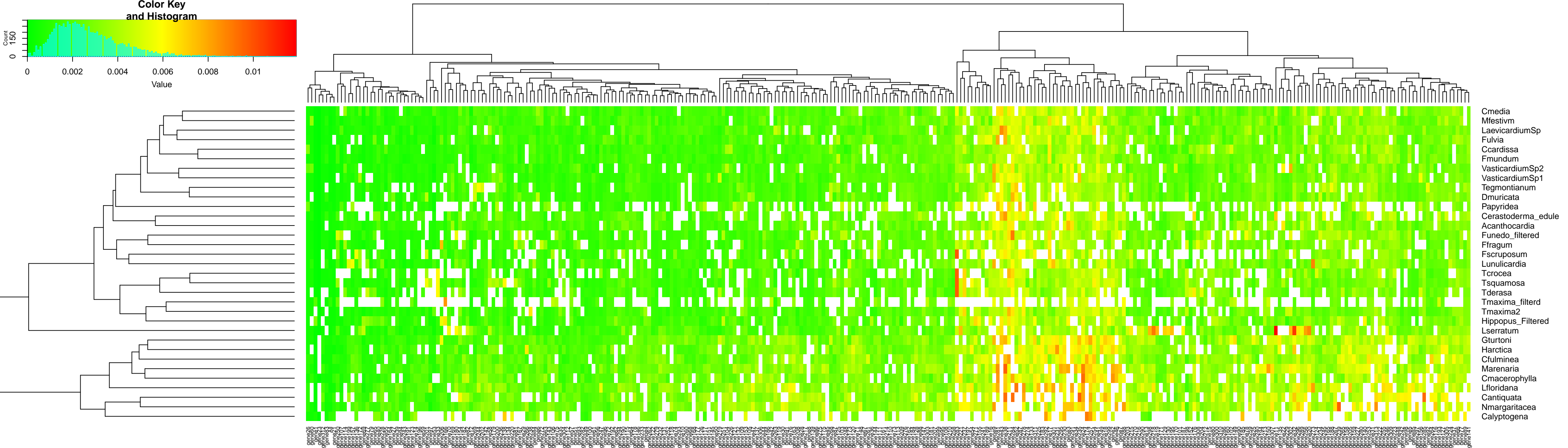

Supplement: Supplementary file 4 — Additional file 4 : Figure S2. A heat map of relative composition frequency variability (RCFV) values for all orthogroups in Matrix 1. Species name are coded as in Additional file 3: Figure S1. [file 12862_2020_1614_MOESM4_ESM.pdf]

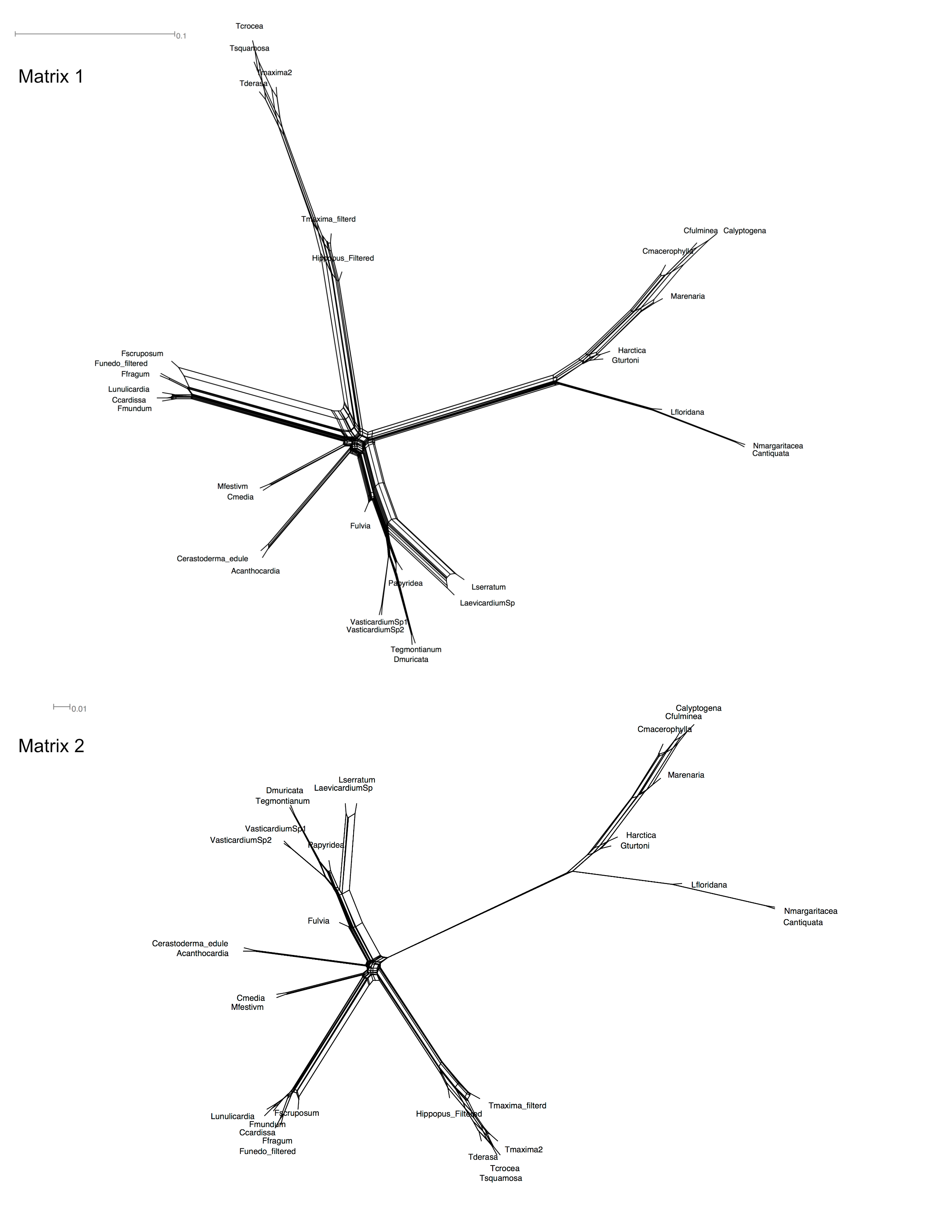

Supplement: Supplementary file 5 — Additional file 5 : Figure S3. Gene super-network for matrices 1 and 2. Species name are coded as in Additional file 3: Figure S1. [file 12862_2020_1614_MOESM5_ESM.jpg]

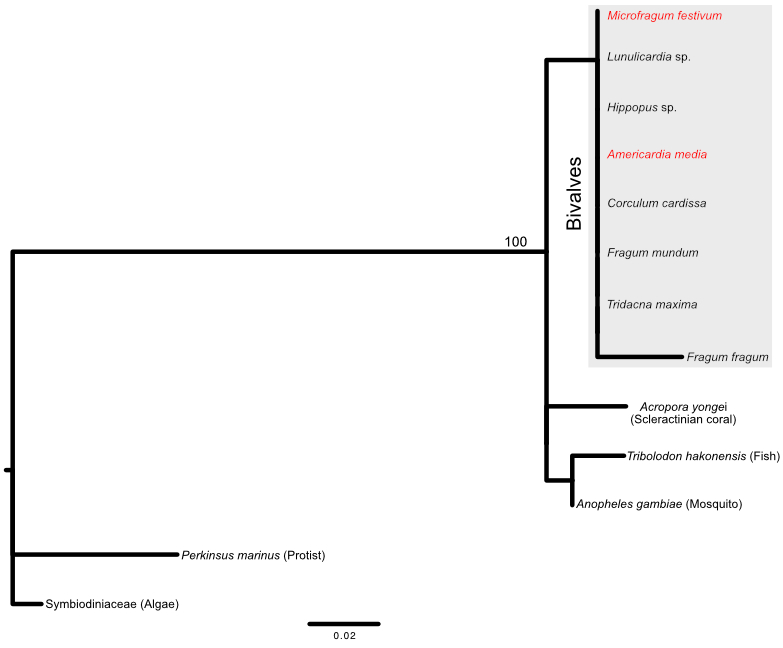

Supplement: Supplementary file 6 — Additional file 6 : Figure S4. Maximum likelihood phylogeny of the VHA genes from Fraginae, Tridacninae, the coral Acropora yongei, Symbiodiniaceae, and other eukaryotes. Non-photosymbiotic bivalve taxa are labeled red. The bootstrap value for the animal VHA clade is shown above the branch. [file 12862_2020_1614_MOESM6_ESM.jpg]
